# Access is Open

**DOI:** 10.1002/vms3.3

**Published:** 2015-03-04

**Authors:** Edward James Hall

**Affiliations:** ^1^ University of Bristol School of Veterinary Sciences Langford House Langford BS40 5DU England

Welcome to *Veterinary Medicine and Science* (VMS), a new, online, peer‐reviewed, Open Access journal covering all aspects of veterinary medicine.

The launch of any new journal is an exciting but uncertain event; uncertain because without any track record (and consequently no impact factor score), why would authors choose such a journal to publish their research? The answer lies both in the perceived benefits of Open Access publishing, and the reassurance that the new journal will be relevant and will publish with integrity. VMS offers that reassurance, backed by an Editorial Board (http://onlinelibrary.wiley.com/journal/10.1002/(ISSN)2053-1095/homepage/EditorialBoard.html) composed of many prestigious veterinary researchers and clinicians.

The benefits of unrestricted access to and reuse of published material are very clear to readers: there is no need to pay a subscription fee to be able to read any paper which has a Creative Commons Attributions (CC‐BY) licence, nor any need to gain permission to reuse the published information (assuming an appropriate attribution is made). Open Access publishing provides an opportunity to enhance discovery through rapid dissemination of new information as well as improving education, particularly in countries which cannot afford journal subscription fees, and providing public enrichment. Indeed Open Access publishing has become the expectation of major grant awarding bodies in the UK, and from April 2016 the Higher Education Funding Council for England will require all UK research to be open access in order to qualify for future funding assessments.

The benefits of Open Access publishing to the submitting author may be less clear as there is a financial cost involved, but there is no doubt it is an expanding medium in veterinary science publication. For example, two of the largest Open Access veterinary journals (BMC Veterinary Research and Acta Veterinaria Scandinavica) published over 350 articles in the 12 months since August 2013. Furthermore, publishing on the Wiley online platform gives the author some extra tangible benefits

- Wiley is a leading veterinary publisher so publication will be robust and permanent
- The article publication charge is very competitive.
- Articles will be read by a wide audience due to the breadth of the journal's subject range.
- Articles will often be highlighted on Wiley's social media feeds and veterinary newsletters.
- Articles will have live citation and download statistics.
- All articles will be given Altmetric (http://exchanges.wiley.com/blog/2014/07/08/altmetric-is-now-on-board-for-all-wiley-journals/;) scores.

VMS welcomes submissions on any topic within veterinary medicine; original work and evidence‐based reviews are equally acceptable. But what VMS will not be is a vehicle whereby any manuscript can be published as long as the author pays. Regrettably some Open Access journals do not exhibit such integrity as was recently exposed by Bohannon (2013), when 157 journals to which he submitted a bogus article containing significant flaws accepted it (often without peer review), whilst only 98 rejected it.

VMS intends to publish high‐quality articles relevant to all areas of VMS, and will establish its integrity through rigorous peer review. As Editor, I am very grateful to an enthusiastic group of Associate Editors who recruit the peer reviewers to make this work. Accepted publications will be of high quality and accessible to anyone with an internet connection.
